# Circular RNAs: Methodological challenges and perspectives in cardiovascular diseases

**DOI:** 10.1111/jcmm.13789

**Published:** 2018-09-11

**Authors:** Matteo Carrara, Paola Fuschi, Cristina Ivan, Fabio Martelli

**Affiliations:** ^1^ Molecular Cardiology Laboratory IRCCS Policlinico San Donato Milan Italy; ^2^ Department of Experimental Therapeutics The University of Texas MD Anderson Cancer Center Houston Texas; ^3^ The Center for RNA Interference and Non‐coding RNAs The University of Texas MD Anderson Cancer Center Houston Texas

**Keywords:** cardiovascular, circRNA, RNA sequencing

## Abstract

Circular RNAs are generated by back‐splicing of precursor‐mRNAs. Although they have been known for many years, only recently we have started to appreciate their widespread expression and their regulatory functions in a variety of biological processes. Not surprisingly, circular RNA dysregulation and participation in the pathogenic mechanisms have started to emerge in many instances, including cardiovascular diseases. Detection, differential expression analysis and validation are the three critical points for the characterization of any RNA, and circular RNAs are no exception. Their characteristics, however, generate several problems that are yet to be completely addressed, and literature still lacks comprehensive definitions of well‐defined best practices. We present a map of the current knowledge regarding circular RNAs and the critical issues limiting our understanding of their regulation and function. The goal was to provide the readers with the tools to critically decide which of the many approaches available is most suitable to their experimental plan. Although particularly focused on cardiovascular diseases, most critical issues concerning circular RNAs are common to many other fields of investigation.

## 1 INTRODUCTION

In the complex landscape of non‐coding RNAs (ncRNAs), a class is emerging as of high biological and functional interest: circular RNAs (circRNAs).

CircRNAs are circularized RNA molecules generated by back‐splicing events on maturing pre‐mRNAs that join together a donor site with an upstream acceptor site. These molecules are therefore expected to comprise exons and introns, although inclusion of intergenic regions has been described.^1^ Despite this heterogeneity, most data are concentrated on exonic circRNAs,^2^ as both early and many recent tools take advantage of known annotation.

CircRNAs possess a number of properties^3^, ^4^, ^5^ that will be enumerated here. They are stable, as they show a median half‐life more than 2.5 time longer than their linear counterparts.^6^, ^7^ They are also resistant to exonucleases, including RNase R, because of the absence of a free tail to initiate the degradation. They show tissue/developmental/age specificity, as their expression is heavily dependent on the cell characteristics. Indeed, circRNAs have been detected with strong specificity regarding tissue of origin, developmental stage and age. They are evolutional conserved, as circRNAs can be conserved as much as their linear counterparts, opening intriguing questions regarding their evolutionary role.

The first circRNAs have been described by Sanger and colleagues in 1976^8^ and subsequently detected in multiple organisms. In mammals, they have been interpreted for a long time as splicing byproducts or errors. Only recently circRNAs have been consistently detected in multiple mammalian tissues—also thanks to the widespread use of deep sequencing techniques aimed towards the detection of ncRNAs—prompting the need of their functional characterization.

Although no generalized function has been identified yet, four main roles have been described^3^, ^4^, ^9^, ^10^, ^11^, ^12^ and are shared by a subset of circRNAs. CircRNAs can work as miRNA sponges. Some circRNAs contain an above‐average amount of seed sequences to bind a specific miRNA and are therefore able to compete for the miRNA and sequester it, reducing its bioavailability and final effect. A key example of this is CDR1AS, a circRNA transcribed in antisense from the CDR1 locus, which contains more than 60 sites for miR‐7. CircRNAs can also act as RNA binding protein sponges. CircRNAs have been observed to interact with RNA binding proteins in order to bind protein complexes and target them towards specific sequences. For instance, Argonaute, RNA Polymerase II and MBNL1 can all bind exonic circRNAs. Moreover, circRNAs can impact gene expression of their host gene locus. CircRNA expression modulation is often correlated with modulation of their linear counterparts, although the mechanism through which this is achieved is still unclear. CircRNAs may bind U1 snRNP and act as *cis* regulator, or just reduce the amount of pre‐mRNA available for canonical splicing. Finally, circRNAs can have a role in translation, as a small fraction of circRNAs seems to contain the necessary information to be translated with a cap‐independent mechanism.

CircRNAs detection experiments identified thousands^3^, ^13^, ^14^ of tissue‐ and condition‐specific species. As of today, no golden standard is available for these kinds of experiments, however, and the differences between the approaches can be significant.

In spite of a number of still open issues, our understanding of the regulation and role of circRNAs is expanding rapidly in all areas of biomedical investigation, including the cardiovascular system. This comes as no surprise, as cardiovascular diseases are among the leading causes of mortality. Indeed, ischaemic heart disease alone accounted for more than 15% of all deaths in 2015 worldwide (WHO Media centre. http://www.who.int/mediacentre/factsheets/fs310/en/.), highlighting the need of a better understanding of the pathogenetic mechanisms underpinning cardiovascular diseases. Additionally, circRNAs are stable and resist degradation, they are present in the bloodstream^15^ and in exosomes,^16^ and therefore, they represent excellent candidates as non‐invasive biomarkers.

The goal of this review was to provide an overview of the approaches used for circRNA investigation. For each method, positive and negative aspects will be illustrated, in order to let the readers critically choose the one more appropriate to their own experimental design. We will also review some of the landmark papers on circRNA regulation and role in the cardiovascular system, paying attention to the methodological aspects adopted in these studies.

## 2 BIOGENESIS

**Figure 1:**
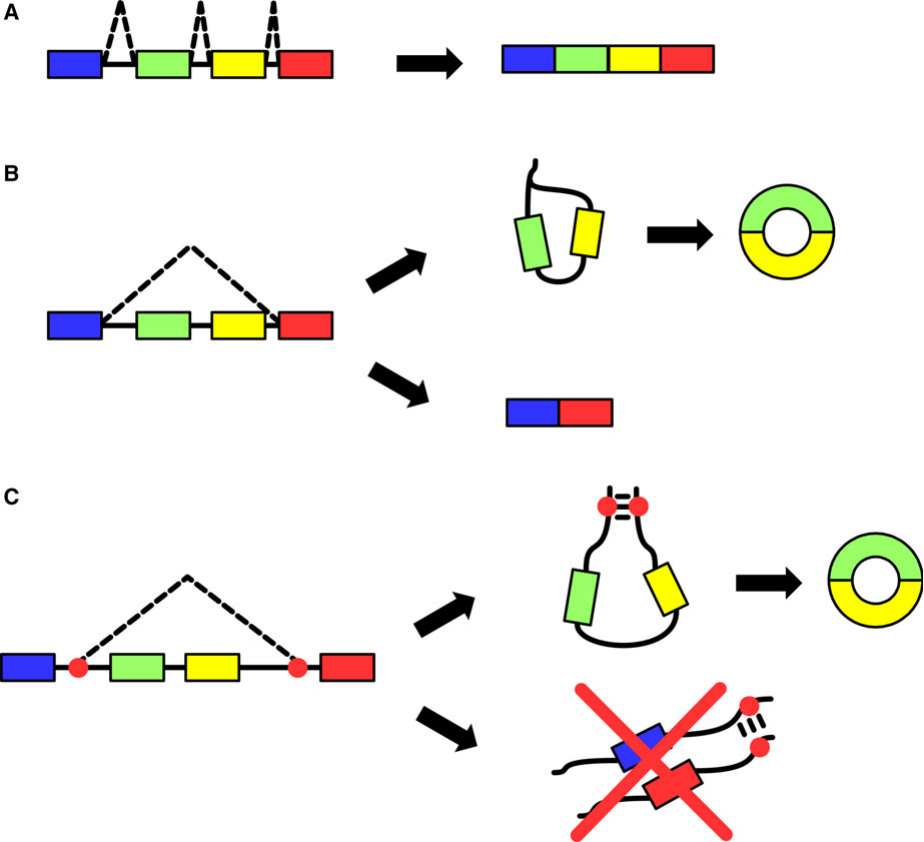
Proposed Mechanisms of CircRNA Biogenesis. A, Canonical linear splicing: Canonical linear splicing determines the maturation of a pre‐mRNA by joining exons together. A donor site and a downstream acceptor site are spliced together, as the introns generate a lariat which is afterwards degraded. B, Lariat‐driven circularization: The linear splicing event takes place first. The lariat generated by the splicing event can then be spliced itself to remove the introns, giving birth to an exonic circRNA. C, Intron pairing‐driven circularization: Alu repeats and other complementary intronic sequences are statistically overrepresented in introns adjacent to back‐splice junctions. RNA molecules are hypothesized to acquire a secondary structure by binding of these sequences and to facilitate circularization

Biogenesis of circRNAs is still an open issue, as studies characterizing the intermediate steps of the circularization events are lacking. Despite that, there are two common models of biogenesis available that can explain the known features of circRNAs, as schematized in Figure 1. The first model is based on lariat‐driven circularization (Figure 1B). Linear alternative splicing commonly produces exon‐skipping events in which the pre‐mRNA is spliced by joining two non‐adjacent exons by the recognition of a downstream branch point.^17^ This event generates a lariat containing the skipped exon (or exons) that can undergo splicing itself. The removal of lariat introns causes circularization of the exon(s) involved. This circularization event is consequence of a linear splicing, and this implies the production of a co‐linear RNA containing the unskipped exons, pairing the expression of each circRNA with the expression of a specific isoform of the linear counterpart. This model has been the first suggested, arising from the evidence of the correlation between circRNAs and their co‐linear counterparts,^18^ a trend not observed in further separate studies.^5^

The second model is based on intron pairing‐driven circularization (Figure 1C). Introns adjacent to the exons involved in the formation of the back‐splice junction have been observed to be significantly longer^19^ and to have a significantly higher concentration of intron motifs and ALU repeats.^17^ It has been theorized that, in the pre‐mRNA, these introns may pair by the interaction between two complementary motifs, promoting a circularizing splicing. This event, although similar to the previous one, does not couple linear splicing and circularization, as a single pre‐mRNA can produce either a circular or a linear species, thus putting the two events in competition for the pool of pre‐mRNA.

Additional hypotheses have been presented based on the biogenesis of aberrant non‐collinear splicing events identified in cancer.^17^ Regardless the specific mechanism, it is critical to remember that the biogenesis might explain expression correlations between circular and linear species, although this relation is not always true.

## 3 DATABASES

The collective effort in circRNA detection using either custom or published pipelines has created the need for a comprehensive database that easily summarizes published events that were predicted, detected or validated.

At the time of writing, we were able to find 11 online databases that include circRNAs, have a web‐based interface and are easily reachable from the links provided in the corresponding papers (Table 1): BBBomics,^20^ circ2Traits,^21^ circBase,^22^ circInteractome,^23^, ^24^ circNet,^25^ circRNADb,^26^ CSCD,^27^ exorBase,^28^ PlantcircBase,^29^ SomamiR,^30^ TSCD.^31^

**Table 1:** circRNA databases

Database	Website	Notes
BBBomics	http://bioinformaticstools.mayo.edu/bbbomics/	Specific for blood‐brain barrier
circ2Traits	http://gyanxet-beta.com/circdb/index.php	CircRNA‐disease associations
circBase	http://www.circbase.org/	Manually curated collection from nine papers
CIRCinteractome	https://circinteractome.nia.nih.gov/index.html	In silico sequence study on circBase data
CircNet	http://circnet.mbc.nctu.edu.tw/	Standalone multi‐library study and manually curated collection
circRNADb	http://202.195.183.4:8000/circrnadb/circRNADb.php	Manually curated collection from five papers
CSCD	http://gb.whu.edu.cn/CSCD/	Cancer‐specific circRNA database
exorBase	http://www.exorbase.org/	Specific for blood exosomes
PlantcircBase	http://ibi.zju.edu.cn/plantcircbase/	Specific for plants
SomamiR	http://compbio.uthsc.edu/SomamiR/	Effects of miRNA somatic mutation on RNA
TSCD	http://gb.whu.edu.cn/TSCD/	Wide standalone multi‐library study

A recent study by Ying Xu provides an overview of the major databases currently available.^32^ The two most widely used are circBase and circNet, but their manual curation slows down the inclusion of the increasing amount of studies published every month on the topic. It is evident that, at present, we lack comprehensive databases that gather all information regarding circRNAs in one common place. To achieve this, a database needs to be generic, structured, updated frequently and partially automated in a fashion similar to Gene Expression Omnibus, RefSeq^33^, ^34^ and miRBase.^34^ The first critical step would be to allow the submission of published detected circRNAs from research teams and to provide a background system to aggregate the submitted data. This ever‐growing, low‐maintenance system can then be used as a base to provide all kind of exploratory and statistical data on circRNAs.

## 4 RNA‐SEQ

### 4.1 Detection

The advent of total RNA sequencing libraries depleted of rRNA opened the door for the detection and analysis of all classes of ncRNAs characterized by the absence of a 3′ polyadenilated tail. CircRNAs are present in such libraries, although canonical alignment methods are not able to detect them, as they rely on the properties typical of linear splicing events. The alignment begins with a seed that is then extended and, when an intron is reached, the alignment is resumed downstream to take into account introns and collinear splicing. Non‐collinear splicing events are characterized by a splicing that may connect one exon with another that is largely distant, upstream or on a different chromosome. All reads supporting a non‐collinear splicing, including back‐splicing, are discarded by classic aligners.

The discovery of non‐collinear splicing events generated a collective effort for the development of pipelines designed for the detection of these events in general and of circRNAs in particular. A comprehensive review by Zeng et al^2^ describes the main circRNA detection tools (Table 2) and evaluates them for precision and sensitivity. While all have merit, the space for improvement is still clearly present for back‐splicing events detection.

**Table 2:** CircRNA detection tools

Tool	Method	Aligner	CircRNAs detected	Website
acfs	Pseudo‐reference	BWA‐MEM	Exonic De novo	https://github.com/arthuryxt/acfs
CIRCexplorer	Fragmentation	BWA, STAR	Exonic Intronic	https://github.com/YangLab/CIRCexplorer2
CIRI	Fragmentation	BWA	Exonic Intronic Intergenic	https://sourceforge.net/projects/ciri/
circRNA_finder	Fragmentation	STAR	Exonic Intronic Intergenic	https://github.com/orzechoj/circRNA_finder
DCC	Fragmentation	STAR	Exonic Intronic Intergenic	https://github.com/dieterich-lab/DCC
find_circ	Fragmentation	Bowtie2	Exonic Intronic Intergenic	https://github.com/marvin-jens/find_circ
KNIFE	Pseudo‐reference	Bowtie, Bowtie2	Exonic De novo	https://github.com/lindaszabo/KNIFE
MapSplice	Fragmentation	Bowtie	Exonic Intronic	http://www.netlab.uky.edu/p/bioinfo/MapSplice
miARma‐Seq	Fragmentation	BWA	Exonic Intronic Intergenic	http://miarmaseq.idoproteins.com/
NCLScan	Pseudo‐reference	BWA, Novoalign	Exonic	https://github.com/TreesLab/NCLscan
PTESFinder	Pseudo‐reference	Bowtie, Bowtie2	Exonic	https://sourceforge.net/projects/ptesfinder-v1/
Segemehl	Fragmentation	(Internal)	Exonic Intronic Intergenic	http://hoffmann.bioinf.uni-leipzig.de/LIFE/segemehl.html
UROBORUS	Fragmentation	Bowtie, Bowtie2, Tophat2	Exonic	https://github.com/WGLab/UROBORUS

CircRNA detection is based on the correct assignment (“recall”) of otherwise not‐mappable reads to putative back‐splice junctions and can be divided into two large categories: segmented read based (or fragmentation based) and candidate based (or pseudo reference based).

Segmented read‐based strategies split unmapped reads in smaller fragments that are aligned separately. The reconstruction of the alignment pattern allows to identify non‐collinear splicing events if the position of the fragments is discordant, that is on different loci, chromosomes, upstream or just in a different orientation. Their nature allows de novo detection and is completely independent from an existing annotation.

Candidate‐based strategies rely on existent annotation and exploit it to generate putative events on which reads are tested for alignment. These tools lack the positive traits of segmented read‐based strategies and can often report only exonic circRNAs, but they tend to be faster in execution and to produce a list of exonic circRNA with a more reliable position of the splice site.

All tools implement a number of filters built to increase precision. The nature and strength of these filters directly correlate with precision and sensitivity, with the tools using the most stringent filters being the most precise and less sensitive, while the tools using less stringent filters tend to trade precision in favour of sensitivity. As suggested for other non‐collinear splicing events,^35^ it is advisable to achieve the highest sensitivity and add biologically meaningful, manually curated filtering whenever possible.

CircRNAs often display low expression levels compared to mRNAs.^13^, ^14^, ^36^ This is particularly crucial in RNA‐Seq datasets, because the most important filter to apply is based on the reads spanning the back‐splice junction^35^ that are only a small fraction of all reads supporting the circular species. This limitation is caused by the inability to uniquely assign a read to either the linear or the circular transcript without additional information. Thus, a strong increase in the sequencing depth is necessary to compensate for the rarity of the information.

Few comparisons between detection methods are available in the literature,^2^, ^37^ apart from the papers describing a new method and comparing it to the major existing ones. Thus, no consensus has been reached regarding the best tool because of the following reasons. There are small differences in the comparison approaches and the tools taken into consideration. There are different definitions for the “best,” that is commonly identified as the most balanced trade‐off between precision and sensitivity. Additionally, different datasets are used for the comparison, and this is critical, as circRNAs show evidence of tissue specificity and high variability between samples.

It is therefore advisable to use different tools, depending on the dataset to be analysed and on the experimental question. All tools work at different efficiencies to balance false‐positives and false‐negatives. Common techniques to reduce one tend inevitably to increase, at least marginally, the other. For this reason, we can identify two lines of conduct: stringent and flexible.

A stringent detection approach requires the use of a tool with high precision and therefore a controlled amount of false‐positives. NCLScan^38^ is a good example. This approach ensures a small list of strong candidates by stringent filtering and by imposing high detection thresholds. The downside resides on the inability to expand the analysis to other biologically relevant events. A list too small can also negatively influence the differential expression analysis. It is suggested to choose this approach when detection and validation are the only aims.

A flexible detection approach relaxes the thresholds in order to become inclusive and detect most true events, increasing also false‐positives. Examples are CIRI,^39^ CIRCexplorer^36^ or KNIFE.^40^ With a balance more on sensitivity than on precision, they provide longer lists of events that may easily reach the level of thousands. Flexible approaches are more suitable for subsequent differential expression analysis, allowing to manually define the selection criteria used to rank the events for validation.

In the landscape of detection tools, miARma‐Seq^41^ fills a different role. miARma‐Seq is a complete pipeline that allows detection of short and long RNAs bundling major detection and differential expression tools in a single package. CircRNA detection is performed using CIRI internally, with similar expected results.

### 4.2 Differential expression

Differential expression for alternative splicing events is a matter only partially solved. Back‐splicing events introduce an additional level of complexity to the problem, a layer that has not yet been properly defined. CircRNAs, in particular, possess a number of critical aspects that influence the differential expression analysis, and we shall list them. (a) Coverage distribution on circRNAs is yet to be modelled. (b) The amount of circRNAs detected in different samples may vary greatly. (c) The number of circRNAs unique for each biological replicate tends to be very high if the detection is performed using tools with low precision. (d) The quantity of reads univocally assigned to each circRNA is usually lower than the one assigned to the linear counterparts. (e) The variability between samples is high. These criticalities make the choice for the most suitable differential expression analysis tool an open problem that many groups have tackled in a “custom” manner, as the literature is still lacking a comprehensive population study of circRNAs expression on which to base the statistics.

It is important to remember that, regardless the tool of choice, so far no differential expression analysis software has been designed to correctly handle circRNAs, thus making any result unreliable. Nevertheless, there are two main tools that can be used for differential expression analysis: limma^42^ and edgeR.^43^ EdgeR method limits the amount of assumptions for the statistical analysis, and it is the method of choice in every occasion in which the expression pattern is unknown and not modelled. The downside of this approach is its sensitivity to variability between samples, a common characteristics in circRNA data.

Limma method was designed for differential expression analysis based on linear models on microarray data and was subsequently used for RNA‐Seq data as well. Of particular interest, it was the introduction of the *voom* transformation, a method explicitly created to increase the power of the analysis by compensating variability. Limma therefore provides the tools to soften one of the heaviest problems in circRNA data, provided the assumption that the expression patterns of circRNAs follow those of mRNAs.

A third alternative is represented by DESeq2, a method allowing quantitative data analysis focused on the strength rather than the presence of differential expression.^44^ However, further tests are necessary to validate its use for circRNA analysis.

The quantification of circRNAs relies on methods that are available and proven for general RNA‐Seq data. The most common and widespread quantification is based on direct counts of features. A specialized software takes as input the aligned reads and compares them against a reference, that is, a list of features of interest with their absolute genomic location. For each feature, the software outputs the amount of reads aligned on that region. As sequencing is a method of absolute quantification, counts can be directly used with a differential expression software and are a representation of the abundance of that feature within the sample.

Direct counting is, however, dependent on the alignment step, by far the most computational‐heavy task of a full sequencing pipeline, because of the need to directly compare billion of bases to the reference genome. The need for faster quantification prompted the development of methods based on “pseudo‐alignments.” Kallisto, Salmon and Sailfish are commonly used quantification tools that completely avoid alignment.^45^, ^46^, ^47^ Briefly, all methods rely on a comparison of k‐mers, that is of chunks of the reads, to be compared using similarity coefficients instead of looking for a perfect 1:1 match base by base. This enables a statistically accurate evaluation of similarity between features and reads, taking into account mismatches—thanks to the similarity threshold—while reducing drastically the run time.

### 4.3 Proposed pipeline

**Figure 2:**
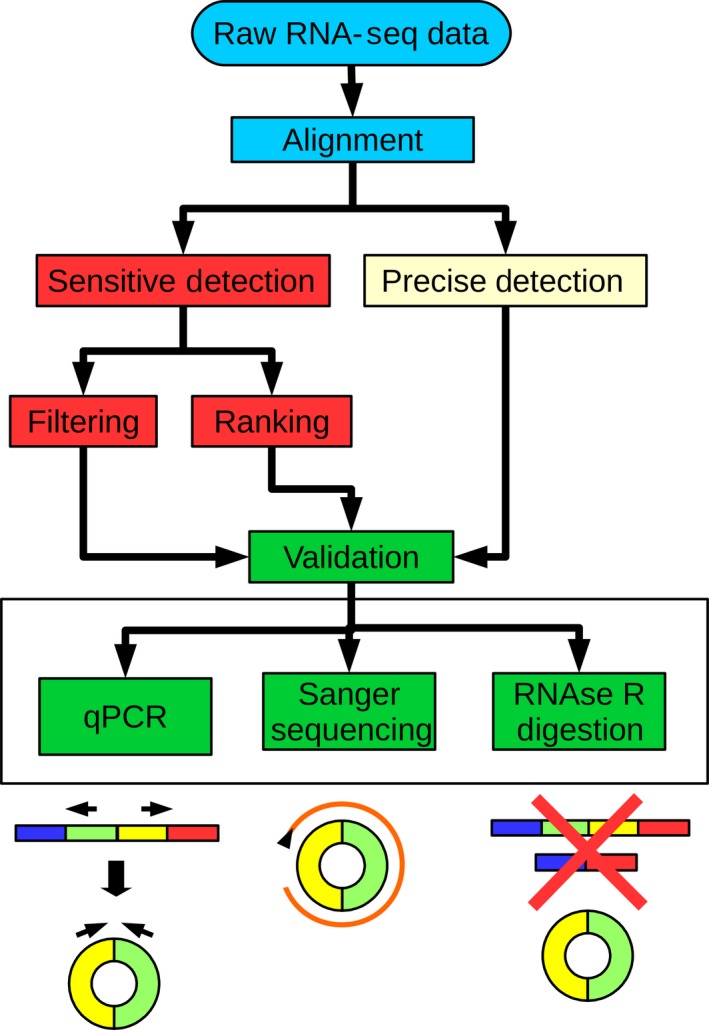
Suggested CircRNA Detection, Differential Expression Analysis and Validation Flowchart. Each level of the flowchart represents a specific passage of the pipeline, with colour code highlighting passages that are part of a unique analysis step. Multiple viable approaches are suggested as branches of a specific level. The canonical starting point is RNA‐Seq data that undergo alignment on a reference genome. CircRNA detection can be performed in a precise or in a sensitive way. As sensitive detection methods are prone to false‐positives, they should be coupled with additional downstream ranking or filtering approaches. The results provide a list of candidates for validation, suggested to be performed with three methods in parallel: qPCR to detect the back‐splice junction, Sanger sequencing to determine the circRNAs sequences and RNase R digestion to confirm the circularity of the events

Differential expression analysis of circRNAs in RNA‐Seq datasets still lacks a fully validated pipeline. Therefore, there are at least three control points which allow freedom of choice that might drastically change the final results: detection, differential expression analysis and enrichment (Figure 2).

Several detection tools exist, and they can be categorized according to their precision and sensitivity. A precise tool is advisable when high‐confidence, highly expressed circRNAs are the objective. At contrary, sensitive tools provide large datasets useful for exploratory studies and enable tentative differential expression analysis.

Differential expression analysis tools are multiple, but, because of the expression levels of the circRNAs, no whole‐transcriptome method is efficient or described as valid. Differential expression analysis is therefore limited to PCR quantification of selected events or normalization and comparison via clustering and heatmap.

Enrichment and validation are more evolved and rely on the combination of multiple methods to confirm position and existence of back‐splice junction, circularity and nucleotide sequence by qPCR, RNase R digestion and sequencing, respectively.

## 5 MICROARRAYS

Microarrays are the election method for detection and differential expression analysis. Arraystar Inc. produces the first commercial microarray that hybridizes specifically on a selected panel of circRNAs (https://www.arraystar.com/arraystar-human-circular-rna-microarray) and was used in milestone studies in the field.^3^, ^13^, ^14^, ^36^, ^48^, ^49^ A number of recent investigations take advantage Arraystar's microarrays and report strong detection and validation efficiency.^50^, ^51^, ^52^, ^53^, ^54^, ^55^, ^56^

Microarray analysis removes the uncertainty that shrouds RNA‐Seq analysis because of a lack of generalization. The procedure is targeted and, when the reproducibility and efficiency are ensured by the manufacturer, the standard analysis methods can be perfectly applied regardless of the hybridized RNA species. It is, however, important to remember that any microarray analysis depends on the annotation accuracy at the time of development and allows only the detection of the annotated RNA species.

Finally, the procedure of labelling requires digestion with RNase R to enrich for circular species. As we will see in the “Validation” section, digestion might introduce biases that are not yet quantified, potentially skewing the differential expression analysis for a small amount of circRNAs.

## 6 VALIDATION

Validation of circRNAs is a multi‐step passage. Initially, the presence of the back‐splice junction must be verified. This can be achieved with a standard qPCR experiment using divergent primers (Figure 2). As described before, a back‐splice junction joins a donor site with an upstream acceptor site, disrupting the canonical linear splicing pattern. Primers positioned around the back‐splice junction and in opposite direction can therefore amplify the region only in presence of a back‐splicing event.

qPCR, however, is not enough to define a species as a circRNA. Lack of linear nature or of free ends can be demonstrated with tests based on resistance to digestion. Circularized RNA molecules (circRNAs and intron lariats) are known to have a long half‐life because of their resistance to degradation. RNase R is a 3′‐5′ exoribonuclease able to degrade linear RNAs. Resistance to this particular RNase has been defined in the past as a major evidence of circularization, and it has been often used as an enrichment method. Unfortunately, it is now clear that resistance to RNase R is not enough to declare an RNA as circular and some circRNA are indeed degraded by RNase R, while certain linear RNAs are, at least in part, resistant. Methods that provide better enrichment have been published.^57^ This inconsistent behaviour complicates reliable quantification and differential expression; thus, digestion is mainly indicated as one of multiple tests for qualitative circularity validation.

## 7 INIHIBITION AND OVEREXPRESSION

Functional characterization of circRNAs requires experiments designed to understand the perturbations caused by the modulation of their expression levels. This is usually achieved by overexpression and inhibition.

**Figure 3:**
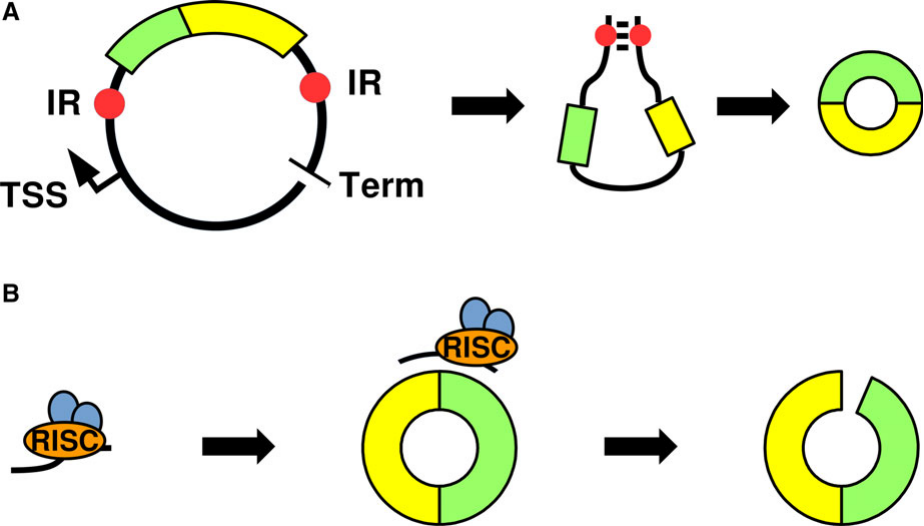
Major Methods of CircRNA Overexpression and Inhibition. A, Overexpression plasmids contain the circRNA sequence (coloured bars) flanked by intronic sequences containing inverted repeats (IR, red dots) and splicing signals (data not shown). The transcribed sequence produces a secondary structure that facilitates the two ends of interest to be spliced together and circularize. TSS, transcriptional start site; Term, transcriptional terminator. B, Inhibition by siRNA requires a treatment with a siRNA complementary to the back‐splice junction. The siRNA, loaded with the RISC complex, is able to selectively bind the circRNA and reduce the available pool of the target molecule by endonucleolytic cleavage

Details have been described in recent reviews by Barrett and Salzman and by Huang et al.^58^, ^59^ Briefly, overexpression can be achieved using vectors containing the circRNA sequence flanked by introns containing inverted repeats and the necessary splicing signals to favour circularization (Figure 3A). The transcription of the vector generates an RNA with introns that can pair and stimulate the circularization. This method, however, is prone to the generation of concatamers if the RNA polymerase fails to recognize the transcription terminator.

The method of choice for inhibiting circRNAs is RNA interference, which uses short interfering RNAs complementary to the sequence around the back‐splice junction. The interaction between the siRNA, loaded in the RISC complex and the circRNA determines the degradation (Figure 3B). SiRNAs built to target the back‐splice junction, however, have the potential to bind partially to and silence the linear counterparts as well, in spite of chemical modifications aimed to minimize this shortcoming.

Additionally, whenever relevant, CRISPR‐Cas9 genome editing technology can be used to remove the locus encoding the circRNA.^60^

## 8 CIRCRNA IN CARDIOVASCULAR DISEASES

As it happened in many other areas of investigation, circRNAs regulation and function in the cardiovascular system have become a research hotspot in the last few years. Although very little is still known, evidence of circRNA implication in heart and blood vessels development, function and disease is clearly emerging. We will review some of the most paradigmatic studies, hoping to stimulate a rapid growth of the field.

### 8.1 Heart function and disease

A landscape of the circRNAs expressed in the heart has started to be defined in broad RNA‐seq projects,^61^, ^62^ as well as in some more focused studies characterizing human, rat and mouse hearts.^63^, ^64^, ^65^

CircRNAs have been shown to be dynamically expressed in a model of cardiac development constituted by human induced pluripotent stem cell‐derived cardiomyocytes.^66^ Sequencing revealed more than 4500 circRNAs, some of which with a host‐gene‐independent regulation. Examples of these circRNAs of interest are those generated from *ATXN10*,* CHD7*,* DNAJC6* and *SLC8A11* genes, which may interact with ribosomes and the RISC complex.

In a circRNA‐microarray profiling comparing neonatal and mature postnatal human cardiac tissue samples, circAMOLT1 emerged as preferentially expressed in neonate hearts.^50^ circAMOLT1 physically binds to both PDK1 and AKT1, leading to AKT1 phosphorylation and nuclear translocation. This, in turn, facilitates AKT cardio‐protective function, both *in vitro* and in a mouse model of doxorubicin‐induced cardiomyopathy.

miRNAs have a major role in the regulation of remodelling and therefore circRNA with sponging capabilities may contribute to this event. Two microarray analyses validated 43 and 45 circRNAs differentially expressed in myocardial fibrosis and in diabetic DB/DB mice, respectively.^51^, ^67^ Of those, circ_010567 and circ_000203 have been shown to act as sponges for miR‐141, a miRNA able to down‐modulate the expression of TGF‐B1.

In a qPCR screening of 100 randomly selected circRNAs, mm9‐circ‐012559, renamed HRCR, was found to be decreased in a mouse model of heart hypertrophy.^68^ CircRNA HRCR can act as a sponge for miR‐233, resulting in the increase in the miR‐233‐target ARC and reducing cardiac hypertrophy and heart failure in mice.

Hypothesis‐driven studies are also important. CDR1AS is a well‐known circRNA, first identified as a miR‐7a/b sponge and inhibitor in brain cells.^3^, ^60^, ^69^ Geng and collaborators found that CDR1AS is also expressed in the heart. In myocardial cells, it acts as a miR‐7a sponge, interfering with the miRNA protective role in myocardial infarction injury.^70^

Ageing is a major risk factor of decreased heart function and a crucial stratification factor for heart‐related diseases. circFOXO3 has been found to be up‐regulated in aged hearts of both humans and mice^71^ and correlates with markers of cell senescence. The functional relevance of circFOXO3 was demonstrated in doxorubicin‐induced mouse cardiomyopathy. In these mice, heart disease is aggravated by the overexpression of circFOXO3 and is attenuated by its silencing. Mechanistically, circFOXO3 interacts with ID1, E2F1, FAK and HIF1A in the cytoplasm, blocking their nuclear translocation, thus inhibiting their anti‐stress and anti‐senescence functions.

RNA‐splicing regulators are of obvious relevance for circRNA biogenesis and modulation. Indeed, important insights came from the study of RBM20, a gene involved in the process of exon skipping in the heart. Of note, its mutation in patients causes a severe form of familial dilated cardiomyopathy.^72^ CircRNA profiling by RNA‐seq of human hearts allowed the identification of 80 circRNAs originating from the *titin* gene (TTN), a gene that is known to undergo highly complex alternative splicing.^73^ A subset of these circRNAs are dynamically regulated in dilated cardiomyopathy and RBM20‐null mice completely lack these titin circRNAs. Specifically, the loss of RBM20 affects only the circRNAs that originate from the I‐band of titin. Thus, RBM20, by excluding specific exons from the pre‐mRNA, might provide the substrate to form this class of titin circRNAs.

Another important RNA‐binding protein that regulates pre‐mRNA splicing is Quaking. The expression of *Quaking* gene is down‐regulated in the murine myocardium exposed to doxorubicin and its deletion increases cardiomyocyte sensitivity to the treatment, while its overexpression blocks doxorubicin‐induced apoptosis.^74^ Of note, Quaking regulates the expression of specific circRNAs derived from a subset of genes, including *titin*, and the inhibition of titin‐derived circRNA increases the susceptibility of cardiomyocytes to doxorubicin.

### 8.2 Endothelium and angiogenesis

A landscape of the circRNAs expressed in vascular cells has started to be defined.^61^

Whole transcriptome analysis of circRNAs in endothelial cells exposed to low oxygen tension was performed by Boeckel et al.^75^ Among the hypoxia‐induced circRNAs identified, silencing of circZNF292 reduced tube formation and spheroid sprouting of endothelial cells *in vitro*. Moreover, circZNF609 was identified among the most abundant circRNAs in endothelial cells.^75^ In an independent paper, Liu et al studied its regulation and function in the retina vasculature.^76^ circZNF609 is significantly up‐regulated upon hypoxia and high glucose exposure in vitro, as well as in patients affected by diabetes mellitus, hypertension and coronary heart disease. Silencing of circZNF609 leads to reduced retinal vessel loss, blocks pathological angiogenesis, increases both migration and tube formation, protects against stress generated by oxidative stress and hypoxia. Mechanistically, circZNF609 is known to be a miRNA sponge for miR‐615, which leads to the up‐regulation of MEF2A.

It is also worth noting that circZNF609 has been identified also in other systems. Indeed, circ‐ZNF609 is necessary for skeletal myoblast proliferation and can be translated into a protein in a splicing‐dependent and cap‐independent manner, providing an example of a protein‐coding circRNA in eukaryotes.^10^ circZNF609 is also expressed in neurons^77^ as well as in bowel tissues, where it is down‐regulated in Hirschsprung disease.^78^

Dang et al also studied hypoxic endothelial cells and, by microarray expression profiling, identified hsa_circ_0010729.^54^ Knocking down this circRNA leads to suppressed proliferation and migration as well as increased apoptosis of endothelial cells. Bioinformatics prediction and experimental validation indicate that hsa_circ_0010729 and hypoxia inducible factor 1 alpha (HIF‐1α) are both targeted by miR‐186. Thus, hsa_circ_0010729 targeting of the miR‐186/HIF‐1α axis underpins its regulation of endothelial cell proliferation and apoptosis.

Moving to other vascular cells, hsa_circ_000595 was identified in a qPCR array profiling study as modulated in hypoxic aortic smooth muscle cells and in aortic aneurysm.^79^ hsa_circ_000595 targets COX2 and NFKB, two proteins strongly correlated to cardiovascular diseases, and acts as a sponge for miR‐19a. Knockdown experiments showed decreased apoptotic rate of smooth muscle cells.

Finally, while most studies investigated circRNA regulation by hypoxia, other stimuli, as expected, are also relevant. A microarray profiling has identified circRNAs modulated in oxidized‐LDL‐treated endothelial cells. Among these, hsa_circ_0003575 has been validated as up‐regulated and correlated with increased proliferation and angiogenesis.^53^

### 8.3 Atherosclerosis

CircRNAs studies in atherosclerosis patients are dominated by circANRIL. This is an antisense circRNA generated by the *9p21* locus, whose SNPs have been linked in GWAS studies to atherosclerotic vascular disease, as well as to type 2 diabetes mellitus and other diseases.^80^, ^81^

The predominant circANRIL isoform consists of exons 5, 6 and 7, and circANRIL is expressed in both healthy and diseased human vascular tissues, as well as smooth muscle cells and monocyte/macrophages, which all play an important role in atherogenesis.^82^ Interestingly, in these cells and tissues, the abundance of the circular species largely exceeds that of their linear counterpart.

Carriers of the coronary artery disease‐protective haplotype at *9p21* show increased expression of circANRIL and decreased linANRIL in peripheral blood mononuclear cells; linear regression analysis indicates that patients with high circANRIL expression develop less coronary artery disease and highest circular/linear ANRIL ratios are found in disease‐free patients.^82^

Mechanistically, circANRIL is able to inhibit ribosome biogenesis, triggering the activation of the p53 pathway, and, thus, increasing apoptosis and reducing proliferation. This, in turn, induces atheroprotection by reducing the proliferation of the cells within the plaque.^82^ Mechanistically, these events are mediated by the binding of circANRIL with Pescadillo homologue 1 (PES1), an essential 60S‐pre‐ribosomal assembly factor, preventing rRNA maturation.

In an independent study in a rat model of atherosclerosis, reduced circANRIL has been correlated with decreased coronary atherosclerosis and reduced apoptosis and inflammatory factors expression, as well as lower endothelial damage.^83^ While further studies are necessary, this apparent contradiction between human and rat data highlights the importance of a close attention to the techniques used to modulate circANRIL *in vivo* and to the targeted cells (endothelial cells vs smooth muscle and macrophages).

### 8.4 Stroke

Microarrays on HT22 mouse neuronal cells exposed to oxygen‐glucose deprivation/reoxygenation shed some light on the circRNAs involved in cerebral ischaemia‐reperfusion injury. Of the 15 differentially expressed circRNAs detected, mmu‐circrna‐015947 has been validated and bioinformatics suggests its key role in the regulation of apoptosis‐, metabolism‐ and immune system‐related pathways.^52^

Genome‐wide bioinformatics analysis identified circDLGAP4, derived from the circularization of exons 8‐10 of the DLGAP4 gene, as a miR‐143 sponge.^84^ circDLGAP4 levels are decreased in the plasma of acute ischaemic stroke patients as well as in a mouse stroke model. circDLGAP4 acts as a sponge of miR‐143 and its decrease results in the inhibition of the miR‐143 target HECTD1. Decreased HECTD1 expression enhances endothelial‐mesenchymal transition by decreasing the expression of tight junction proteins, with concomitant increased expression of mesenchymal cell markers. Accordingly, circDLGAP4 overexpression significantly inhibits endothelial‐mesenchymal transition and protects the blood‐brain barrier integrity in the mouse stroke model.

### 8.5 Biomarkers in cardiovascular diseases

As previously stated, circRNAs possess strong biomarker potential and many studies aim to characterize new markers for risk stratification and early detection of diseases.^85^

CircRNA MICRA associates with heart failure after acute myocardial infarction.^86^, ^87^ It allows to predict the development of heart failure and enables risk stratification. Of note, MICRA also originates from the *ZNF609* locus, although different exons are involved compared to circZNF609 described in the vasculature,^75^, ^76^ as well as in muscle and neuronal cells.^10^, ^77^

In the plasma of coronary heart disease patients, microarrays detected 24 differentially expressed circRNAs. Bioinformatics analysis generated an interaction network mediated by hsa‐miR‐130a that involves TRPM3 and 9 circRNAs.^88^ The 9 circRNAs are described as sponges for miR‐130a and therefore able to indirectly cause the up‐regulation of TRPM3.

Finally, atherosclerotic plaque rupture is accompanied by an acute decrease in the carotid plaque expression of miR‐221.^89^ CircR‐284 is a potential inhibitor of miR‐221 activity and serum circR‐284/miR‐221 ratio displayed to be a potential diagnostic biomarker of carotid plaque rupture and stroke.

## 9 CONCLUSIONS

The attention of the scientific community to circRNAs is growing exponentially as their involvement in the regulation of both homoeostasis and disease is emerging. They introduce an additional level of control, modulating, either directly or indirectly, a variety of cellular functions and pathways.

Detection methods are still at their early stages and are dominated by biology‐driven approaches or microarray analysis. Next generation sequencing analysis pipelines show the potential to enable transcriptome‐wide detection of circRNAs, but, at present, they need a careful preparation of the experimental plan in order to be reliable. Validation of circRNAs is mostly concentrated on proving the circular nature of the molecule and the presence of the back‐splice junction.

Despite the limitations, circRNAs have an immense potential as therapeutic targets and stable biomarkers, being correlated to risk and developmental stage of many different conditions, including cardiovascular diseases.

circRNAs are far from being completely understood, but they have already proven to be an important field of investigation for clinical and basic research alike.

## CONFLICT OF INTEREST

The authors state that there are no conflicts of interest.
